# Cost-Free LTC Model Incorporated into Private Pension Schemes

**DOI:** 10.3390/ijerph18052268

**Published:** 2021-02-25

**Authors:** J. Iñaki De La Peña, M. Cristina Fernández-Ramos, Asier Garayeta

**Affiliations:** 1 Faculty of Business and Economics, University of the Basque Country, 48015 Bilbao, Spain; asier.garayeta@ehu.es; 2 Department of Education, Junta Castilla-León, 47011 Valladolid, Spain; mcrisferra@gmail.com

**Keywords:** ageing, dependency, long-term care, private pensions

## Abstract

Long-term care coverage is not integrated into an individual’s retirement strategy. It is an additional public health service that is not considered into private pension funds. Nevertheless, this coverage is not sufficient due to the problems of financial sustainability of the public pension systems. However, there are large sums in pension plans dedicated to paying retirement pensions that can be transformed into support for long-term care coverage. This paper develops a mechanism of pension transformation through the different mortality of the beneficiary when becoming a dependent beneficiary. This mechanism allows the beneficiary to convert their pension to LTC support at their own choice, without increasing the cost of the private pension scheme. The proposed model provides consistency in the pension that a retiree receives and adapts it to a retiree’s life expectancy: the retiree receives a higher pension when he/she needs it most.

## 1. Introduction

This paper proposes a mechanism for transforming the old-age pension into a benefit to help pay for long-term care (LTC) within a private defined benefit (DB) pension scheme, should a beneficiary need it, if they become dependent. This action is in line with the World Bank’s proposal, which includes a “fourth pillar” for dependent care, based on family or informal transfers [1]. In turn, it includes one of the greatest challenges of modern society characterised by an increasingly ageing population. It is therefore necessary to develop mechanisms to facilitate LTC services [2].

LTC are those expenses dedicated to the care of older people over a period of time [3]. Such care is provided either in support of activities of daily living (bathing, dressing, eating, getting in and out of bed, grooming and continence) or with instrumental activities of daily living (preparing meals, cleaning, washing clothes, taking medication, walking, shopping, managing money and using the telephone or Internet). Among other causes, LTC mainly arises from the loss of autonomy in old age [4]. To put this in perspective, total LTC expenditure in the United States in 2012 was USD $220 billion (8.7 per cent of total health care expenditure [5]). In addition, between one-third and one-half of retirees in the United States needed care and nursing services, and 10–20% of them required this level of care for more than five years [6]. 

It should be planned well in advance, as should retirement, [7,8] because in most countries the elderly population will increase and family care will decrease due to both demographic and social changes [9,10,11]. Therefore, resources will need to be provided to address this.

Family support, although important, is unable to cope with imminent expenditure, either through direct care by relatives or through inherited legacies from parents to children [12,13,14,15,16]. On the other hand, although public insurance attempts to cover the services demanded [17], it is conceivable that it cannot cope with all the increased expenditure because of the implication for the financial sustainability of the public pension system [18,19,20].

In addition to the public system under the first welfare pillar, there are other collective systems that can help to complete this “fourth pillar” [21,22,23,24,25,26], such as occupational pension funds or funds derived from the second or professional pillar (Table 1). The accumulated funds are large. However, there are no studies that have analysed the possibility of using these pension funds for other needs such as LTC. This paper focuses on the transformation of these pension funds in order to address LTC. Thus, the transformation will allow the beneficiary to choose the type of benefit they want—either through a service or by paying money to finance it. 

The transformation of the private pension fund also implies that LTC coverage should be integrated into an individual’s retirement strategy [3] rather than being considered an additional health service [28]. This is a logical extension of the purpose of pension schemes: to provide an adequate financial complement to meet retirement needs, regardless of an individual’s health status. The design of a pension plan must therefore consider potential LTC needs [29]: On retirement, an income to compensate for the lack of salary, and subsequently, in the appearance of LTC expenses, resources to meet them. Thus, as the population ages, the needs and services demanded change; and when the baby boom generation ages, the more expensive LTC will be needed [30].

The aim of this paper is to develop a pension scheme model that takes into account the future need for both retirement and LTC under an information asymmetry with respect to an individual’s health conditions. The gap that is addressed is to include a mechanism that relates the private pension received to the health status of a beneficiary. This mechanism adjusts the private pension amount to an individual’s life expectancy without the need for additional funding. The individual in the model thus enjoys good health and does not perceive that they need LTC. Such care is only necessary once old age is reached. However, at the time of establishing the retirement savings vehicle, they do not know what their future state of health will be nor what the risk of living to an advanced age will be. Rather, an individual is only aware of the survival probability of reaching retirement. Thus, the main contribution is that a private pension plan model includes a mechanism without increasing the total cost. This strengthens the “fourth pillar” proposed by the World Bank by covering LTC in a sustainable way without placing an additional burden on public pension systems. 

This work’s structure is as follows. Section Two deals with a review of the literature on LTC coverage. An individual’s choice of a defined contribution (DC) pension plan over a DB pension plan is not addressed as it is not the subject of this article. In the third section, both the pension plan model and the mechanism relating the pension amount and life expectancy are included in the methodology and within the model. The fourth section provides a numerical illustration of the model presented in the third section, based on LTC coverage in Spain to illustrate its operation. Section Five addresses the discussion and implications of the model and concludes with future research and limitations. 

## 2. Literature Review

After defining LTC as the cost of providing services to facilitate the range of activities mentioned above, coverage should be considered—from family benefits, income, housing, to daily living costs. The good news is that, in general, the need for LTC only arises at a later age and only for a part of the population aged over 80 [31]. However, an individual is faced with uncertainty about what the true costs of LTC services will be. There is considerable literature on this [32], especially regarding savings and retirement planning decisions.

The design of LTC private coverage [33] starts by defining the daily living costs either through services [34] or benefits (in the form of capital or income) or by combining both [8], both of public and private origin [35,36]. An individual is even given the freedom to decide the kind of care to be financed [37,38,39]. In fact, individuals prefer to use professional services provided by either nursing homes or informal carers rather than family support [40,41]. Although the family has historically been responsible for LTC, the change in family structure, the rapid growth of the elderly population and the development of specific medical services [42,43,44] has relegated it. It should be mentioned that by 1968, LTC coverage had already been introduced in the Netherlands. Its aim was to relieve families and private charities of the financial burden of caring for frail, elderly and disabled relatives. This prompted a rapid expansion of nursing homes, rest homes and institutional care [31].

There is a growing trend toward cash coverage, i.e., providing financial compensation to LTC as in Australia, Canada, Europe, and the United States [45] rather than just guaranteeing a service. A set of measures is normally offered—instead of services—that fully satisfies a dependent person’s needs [46], such as the use of their residence [9], which provides higher satisfaction levels and improved control for the dependent person.

Following the classification of [38], three public coverage models can be distinguished:

1. Provision of services in kind (Netherlands and Sweden).
2. Financial benefits for comprehensive coverage plans (France).
3. Cash benefits for dependent care (Austria, Germany and Italy).

Although it can be considered an extension of health insurance, dependency is a contingency that must be considered at the same time as retirement [28] so that it can protect the insured from the risk of surviving on their available resources after retirement [47]; this is the LTC risk. In this sense, it is proposed to relate the dependency coverage to retirement pensions to extend their effect [48,49,50,51]. Previously, [52] proposed that a dependency insurance be included within the public pension system, introducing an improved pension financed with contributions deducted from the public retirement pension. 

In the private field, natural coverage is already separated from that of LTC [48,53], alleviating the problems of dependence with a product that is adequate to their demand. The combination of different benefits [54] simplifies and integrates an important aspect that is usually dealt with separately in retirement plans: the recognition of a potential need for care due to dependency. Its inclusion leads to a higher benefit in case a beneficiary becomes dependent. This approach was posited by [55] and [56] as a combination of a retirement income and a higher income at the point of dependency.

There are authors who claim that dependency coverage is integrated into retirement planning [57] so that the probability of becoming dependent would already be included in the income to be received. Others, however, claim that dependency and mortality as an independent person are negatively correlated [49,58], thus creating a natural selection of demand for each product. There is no doubt that factors affecting this are age and state of health, there being a degree of uncertainty as to when an insured individual will become dependent [59], although there is evidence of advantageous selection in the sense that individuals who are more reluctant to risk take better care of themselves, live longer, and are more likely to take out income [60] and LTC cover [61].

On the other hand, people living longer are more likely to use long-term care [30,62,63]. Moreover, as an individual ages, the total LTC cost also increases with age, as highlighted by [30,64,65,66]. However, an individual does not appear to plan for LTC in the long term, and according to [67], this may be due to a misperception of risk. Living longer equals a higher current value of LTC, but the needs of the old-age pension would be met by those probabilities. Rather than considering different probabilities of needing LTC, it therefore seems appropriate to incorporate a mechanism that transforms the old-age pension according to the severity of loss of autonomy [68].

The problem lies in the financing, which, as mentioned above [52,56], is obtained by deducting the corresponding premium from the retirement pension. In contrast to this approach, other authors [69,70] propose an approach that integrates LTC into the coverage, which adapts a beneficiary’s pension to their dependent state by using an actuarial correction or reduction factor [71] usually used for early retirement. This approach is simpler and is used in public social provision to relate the pension to pensioners’ life expectancy [72]. In countries such as Switzerland, Poland, Latvia and Norway, a system in which individuals receive a benefit according to their estimated life expectancy and contributions is employed. In this way, individuals from different cohorts will receive a similar return on their contributions [73]. 

It is at this point that this paper proposes a step forward: given the uncertainty of when an individual becomes severely or highly dependent and their priorities thus change regarding their resources to finance LTC, the pension model establishes a factor which automatically transforms a beneficiary’s retirement pension at the moment of becoming severely or highly dependent, and which also considers the mortality differentials of both independent and dependent beneficiaries. This is the paper´s main contribution, becoming a cost-free pension model without adding new financial resources and adapting the private coverage of a pension plan to a pensioner’s resource needs.

## 3. Methodology

### 3.1. The Model

The model is based on the premise that an individual is part of a DB pension plan and lacks information about their future health status. Therefore, they will have a contribution history independent of their future state of health because it depends solely on their career. Furthermore, any additional information about their actual state of health that arises over time does not really affect the pension at retirement. Conversely, if the plan is DC, an individual gains information about their health status and can therefore hire a savings vehicle appropriate to their retirement or LTC needs [74,75,76,77]. This paper develops the initial model proposed by [69,78,79] by including it within a pension plan and adapting the pension to individuals’ LTC needs due to higher degrees of dependency. 

The classification of the degrees of dependency depends directly on institutional factors in each country, with higher LTC being common to all of them as the degree is higher. Lower degrees of dependency may in fact lead to disability pensions. However, dependent people’s mortality is proportional to both the level of care needed and their age, as derived from the study of populations in the UK [80,81] and Norway [82]. The main requirement is therefore to focus on the calculation of the probability of a dependent person’s death that limits the duration of LTC payments.

Therefore, let *X* be the random variable “age of death of a new born”. The death distribution function is represented by *F:*

(1) $$F\left(x\right)=P\left(X\leq x\right)$$

where $x\geq 0\;F\left(0\right)=0$.

In contrast, the survival function is that which for each age *x* provides the probability that a new born will reach that age alive. This is ∀ x≥0,

(2) $$s\left(x\right)=P\left(X>x\right)=1-F\left(x\right)$$

The derived function $f\left(x\right)$ of the death function $F\left(x\right)$ it turns out,

(3) $$f\left(x\right)=\frac{dF\left(x\right)}{d\;x}=-\frac{ds\left(x\right)}{d\left(x\right)}=-\overset{´}{s}\left(x\right)$$

The mortality rate is instantaneous,

(4) $$\mu_{x}=\frac{f\left(x\right)}{1-F\left(x\right)}$$

Like $\mu_{x}\geq 0$ and $f\left(x\right)=-\overset{´}{s}\left(x\right)$ then,

(5) $$\mu_{x}=-\frac{\overset{´}{s}\left(x\right)}{s\left(x\right)}=-\frac{d\;Ln\;\left(s\left(x\right)\right)}{dx}$$

The probability of a person of age *x* reaching age *x + t* can therefore be defined as

(6) pt x=e−∫xx+tμxdz

Likewise, $v_{T}$ is the corresponding financial update factor from the *t-*th instant to the origin or initial moment, the financial update function being defined by the update process to the instantaneous interest $\delta \left(t\right)$,

(7) $$v_{T}=e^{-\int_{0}^{T}\delta \left(t\right)dt}$$

The current compensation value corresponding to the *t*-th moment will be

(8) $$Z_{T}=b_{T}\cdot v_{T}$$

which will also be a random variable as both magnitudes $b_{T}$ and $v_{T}$ depend on the random variable of life-to-death time. 

If the payment function is known as ($b_{T}$), survival as ($s\left(x\right)$) and the financial update as ($v_{T}$), the actuarial value of retirement pensions can be calculated (assuming a duration of the operation from retirement age *r* to age *w* or maximum life expectancy) at age *r* ($PVFB_{r}$), such as

(9) $$PVFB_{r}=E\left(Z_{T}\right)=E\left(b_{T}\cdot v_{T}\right)=\int_{r}^{w}b_{t}\cdot e^{-\int_{r}^{w}\mu_{x}dt}\cdot e^{-\int_{r}^{w}\delta \left(t\right)dt}\cdot dt$$

When a beneficiary of a benefit from a private pension scheme becomes severely or highly dependent at an intermediate age *x*, between age *r* and age *w,* it is assumed that the pension amount is automatically increased by a certain percentage $\lambda_{x}^{d}$ which helps pay for LTC. At each age there is a different factor that is affected only by the different probabilities of death of an independent and dependent individual.

$b_{x}$ is the retirement pension corresponding to age *x*. When becoming a dependent person that amount is multiplied by $\lambda_{x}^{d}$ and this automatically increases the pension by that factor to provide additional resources to help pay for LTC costs. 

### 3.2. The Factor

There is no additional funding, but there is a transfer of the retirement benefit value to the LTC benefit. Therefore, at an age *x > r,* such that a pensioner who is already dependent decides to transform their retirement pension so that there will be equivalence with the present value of future LTC benefit ($PFLTC_{x}$).

(10) $$PVFB_{x}=PFLTC_{x}$$

This is the transformation factor at each age. It depends on the sum of the residual survival probabilities in future years, according to a beneficiary’s status, i.e., life expectancy according to their status (independent or dependent).

(11) λxd=∫xwe−∫tt+1μxdt·dt∫xwe−∫tt+1μtddt·dt=exme dxm

$e^{-\int_{t}^{t+1}\mu_{t}^{d}dt}$: The survival probability of a dependent person aged *t* reaching age *t + 1* as a dependent person.

$e^{-\int_{t}^{t+1}\mu_{x}dt}$: The survival probability of an independent person aged *t* reaching age *t + 1* as an independent person.

$e_{x}^{m}$: Life expectancy of an independent person aged x.

e dxm: Life expectancy of a dependent person aged x.

The value of the resulting aid will depend on the age of the decision, a cohort’s expected mortality, a dependent person’s expected mortality and the level of retirement pension due to a retiree. Except for the expected mortality of a dependent person, all the other factors are common in the design of a private pension plan.

In the actuarial literature on the mortality of insured dependent people, there is unanimity that dependent people’s mortality rates —q dxm—are different from and higher than those of overall mortality —$q_{x}^{m}$—which the standard tables used by insurers for normal risk assessment indicate. They are, of course, significantly higher than the mortality of independent insured persons —q axm—[83]. The following is therefore accepted:

(12) q dxm>qxm>q axm

With these probabilities, the life annuity of a severely or highly dependent person is obtained.

**Figure 1:**
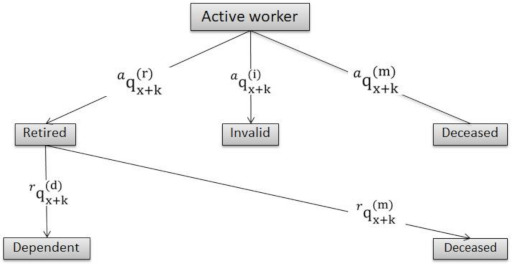
Transition probabilities. Source: Own work.

This paper initially starts from a simplified type of multistate transition model [55] in which the probabilities between various states are described: active to retired (both independent and dependent), to disabled and to deceased (Figure 1). It is a discrete multi-state model for an annual period, where it is assumed that only one transition can occur per year and there is no return to previous states.

Being

p ax+ka: Probability of an active worker aged *x + k* reaching age *x + k* + 1 as an active worker.

q ax+ki: Likelihood of an active worker aged *x + k* becoming disabled before reaching age *x + k* + 1, also being exposed to other causes (death and retirement).

q ax+km: Probability that an active worker aged *x + k* will die before reaching age *x + k* + 1, also being exposed to other exits (disability and retirement).

q ax+kr: Likelihood that an active worker aged *x + k* will retire before reaching age *x + k* + 1, also being exposed to other causes (death and disability).

Where *x + k* is less than the retirement age (*x + k < x < r),* the following equivalence is obtained:

(13) p ax+ka+q ax+ki+q ax+km+q ax+kr=1

which is true for the entire period of activity. 

From retirement age (*x > r*), an individual receives a retirement pension if they are alive. If a retiree dies, they will not receive it, nor will they if they are dependent and decide to convert it to receive LTC assistance. Therefore,

(14) p rx+kr+q rx+km+q rx+kd=1

p rx+kr: Probability of an old-age pension beneficiary aged *x + k* reaching age *x + k* + 1 being retired.

q rx+km: Probability of an old-age pension beneficiary aged *x + k* will die before reaching age *x + k* + 1, also being exposed to other exits (dependency).

q rx+kd: Probability of a retirement pension beneficiary aged *x + k* will become dependent before reaching age *x + k + 1*, also being exposed to other causes (death).

This probability is considered to be a binomial value (0; 1), where it takes a value of 0 when a beneficiary decides to continue receiving their retirement pension and takes a value of 1 when a retiree decides to transform it to help with LTC expenses. With the above, it only remains for a dependent person to be determined:

p dx+kd: Probability of a retirement pension beneficiary who is dependent aged *x+k* reaching age *x + k + 1* while being dependent.

q dx+km: Probability of a retirement pension beneficiary who is dependent aged *x+k* to die before reaching age *x + k + 1*.

Where obviously its sum is the unit at age *x + k*,

(15) p dx+kd+q dx+km=1

If in this model a factor $\lambda_{x}^{d}$ is applied when becoming a dependent person, only the probability of death as a dependent person should be determined. Studies have been conducted in which the probabilities of suffering severe and high dependency have been determined [79] and from which the life expectancy of an individual suffering the most severe stages of dependency have been established. In those studies, starting from a general mortality, dependent people will have an excess mortality that can be expressed by a multiplicative correction-θ:

(16) q dxm=θ·qxm

This correction can be variable at each age, although [84] indicated that a fixed correction adjusts the mortality of older dependent people better than other types of approximations. However, it tends to overestimate the mortality of dependent people at lower ages and to underestimate it at higher ages. Taking this into account, it is more accurate to make an additive adjustment —ε— to the overall mortality by considering age as an independent variable in a functional form [85]:

(17) q dxm=qxm+ε where ε=fx

The result is that mortality rates increase with the level of dependency, being lower at younger ages; for less severe dependency, no excess mortality is applied [86].

## 4. An Application to Spain Results

LTC institutional systems organise dependency in degrees of severity, from the mildest to the most severe, depending on the number and kind of activities of daily living an individual can perform. This classification has a direct impact on the public aid received; both the classification and the amount of aid varies in each country. In Spain, there are three levels of dependency:

(a) Level I: Moderate dependency. The individual needs help to perform several basic daily activities at least once a day or has intermittent or limited support needs for personal autonomy.
(b) Level II: Severe dependency. Assistance is needed to perform several basic daily activities with a frequency of two or three times a day, but does not require permanent assistance.
(c) Level III: Severe dependency. Assistance is needed to perform several basic daily activities several times a day. Due to total loss of physical, mental, intellectual or sensory autonomy, the individual needs extensive support for personal autonomy.

For these levels, the administration establishes three different coverages:

(i) Basic: Essential coverage is provided and financed by the Spanish General Administration.
(ii) Complementary: the coverage is provided by agreements between the Spanish General Administration and the Autonomous Communities.
(iii) Improvement Level: the private sector has a role in this area.

The private sector almost exclusively offers coverage for the highest levels of dependency [87], either through insurance or through pension plans. In fact, dependency was added [88] to the contingencies of a pension plan, although it was contemplated in the levels of severe or high dependency. In this way, DC pension plans allow individuals increased freedom to invest their funds to meet LTC expenses. In DB schemes, however, consideration of dependency coverage leads to more profound changes. The defined benefit implies voluntary collection as well as redefining the benefit amount through a pension conversion factor. 

### 4.1. Severe and Highly Dependent Mortality

Reference [82] determined the probability of death of severely and highly dependent from Spanish mortality tables fitted to Handicap-Incapacity-Dependence (HID) 98-01 statistics for France.

They noted that the differentials in excess mortality with respect to overall mortality decreased from the age of 96. To reflect this effect, they included a variation of the [86] formula from a mixed correction on general mortality to model dependent mortality. In this mixed correction, an additive modification was considered under the expression of the [86] formula and a multiplicative correction on the general mortality scores that reflected the decrease in the absolute mortality differentials in older ages of the table. The function is:

(18) q dxm=qxm+δ1+γxi−x ∀xi<95qxm·1+β+δ1+γxi−x ∀xi≥95

δ: Maximum value to be incorporated according to the age at which it asymptotically converges.

γ: Slope factor.

$x_{i}$: Age at the inflection point where the curve changes shape from convex to concave.

β: Multiplier factor on overall mortality.

**Figure 2:**
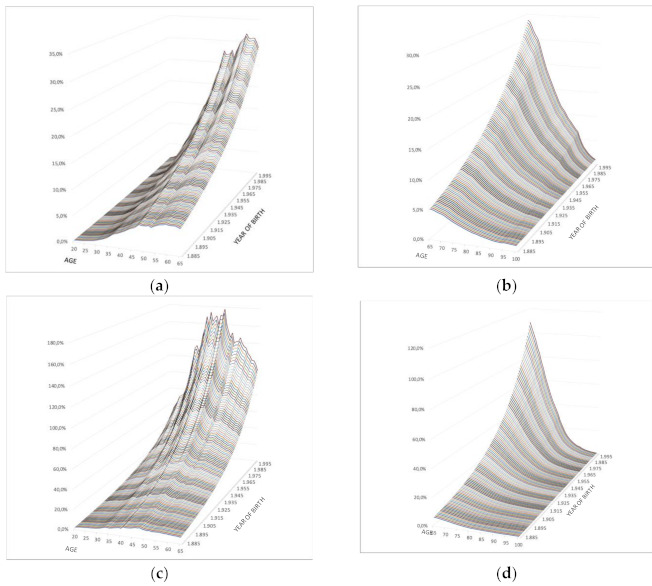
Increase in the mortality rate due to dependence in Spain. Evolution by gender and age: (**a**) Men, period of work activity from the age of 20 to the age of 65, according to year of birth; (**b**) Men, period of retirement from the age of 65 and until the beneficiaries’ death, according to year of birth; (**c**) Women, period of work activity from the age of 20 to the age of 65, according to year of birth; (**d**) Women, period of retirement from the age of 65 and until the beneficiaries’ death, according to year of birth. Source: Own work.

The values obtained with a procedure of ordinary least squares with respect to the gross values of high dependence estimated for Spain are found in Table 2. Mortality rates for severely and highly dependent people are higher than the general mortality rate for all ages (Figure 2). 

Table 3 shows the initial data and mortality values for the dependent born in 1970. The general mortality is based on the Spanish insurance market table PE2000NP. The values of mortality rates by sex and life expectancy for that generation are included. These initial values determine the factor by age, sex and generation of birth. 

For the 1970 generation, the main results obtained by age and sex are shown in Table 4. These results are the factor itself, as well as an analysis of the increase in mortality resulting from becoming a dependent, the reduction in the number of payments due to higher mortality, as well as the differential mortality of dependents at each age by sex.

According to the previous data and results for all the generations, Figure 2 shows the excess mortality among men and women—mainly from the age of 35—by activity and retirement brackets. It can be seen that:

1. The greatest increase in the dependent population mortality over the independent population is found at ages close to retirement, both for men and women, with more pronounced values in more recent generations.
2. During the retirement period, the excess mortality of severely and highly dependent people is reduced to almost zero differentials. This implies that the mortality of a self-employed person is similar to that of a severely dependent person at the end of life.
3. The older generations show lower increases in mortality in dependency than the younger generations, so the effect of severe and high dependency will be significantly greater in the younger generations.

### 4.2. Corrective Factor for Severe or High Dependence 

**Figure 3:**
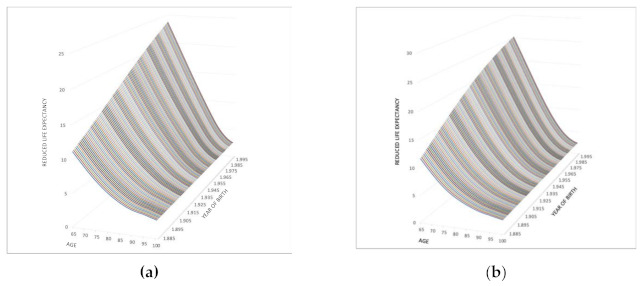
Reduction in the number of payments, according to the age of the severely dependent retiree by year of birth. Evolution by gender and age. (**a**) Men; (**b**) Women. Source: Own work.

A beneficiary will receive their retirement pension according to the expectations of payment calculated according to the state of health. However, a change in their status will not result in a change in the accumulated capital but rather in the way the pension is received, since the priority will not be to replace their salary but rather to help with the expenses incurred by LTC. The expectation of payment under the new circumstances will therefore be affected, leading to a reduced number of payments according to the reduction in life expectancy. In this retirement period, for both men and women, the reduced number of payments will be smaller the older you become and when you become severely or highly dependent (Figure 3).

If it is considered that the highest percentage of people who suffer from dependency are those over 65, the pension calculation should also consider life expectancy as both an independent and dependent person, because otherwise the life expectancy of the elderly would be overestimated. The reduced number of payments means that, for its application, the pension is increased to almost triple, depending on the age at which a person becomes severely dependent. The inclusion of the correction factor therefore takes into account both the expected number of payments and their intensity.

**Figure 4:**
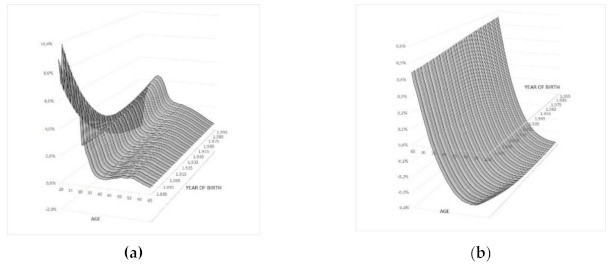
Differential of dependent mortality by gender at each age. Evolution by year of birth: (**a**) Period of activity; (**b**) Period of retirement. Source: Own work.

Taking into account the gender of a beneficiary, the change in the death of women over that of dependent men in the Spanish population stands out (Figure 4). Thus, at ages prior to retirement age and in general (Figure 4a), mortality as a dependent corresponding to women is higher than that of men. However, in the first years of working activity and in generations of advanced age, there is a higher mortality rate which has been decreasing as the year of birth has become more recent—lately it has been even lower than the dependent men mortality rate. This situation changes at retirement age (Figure 4b), where this differential decreases with age and ends up with a dependent mortality rate that is lower than that of older men.

## 5. Discussion

A dependent person seeks to reduce the burden of expenditure due to their health status. Therefore, once dependent, a retiree substitutes their priorities by having higher LTC expenses. Through the corrective factor, a beneficiary will then have more resources to deal with these new expenses, although:

- The increase may be intended to reimburse some of the LTC expenditure rather than being paid directly to a beneficiary.
- If there were a surplus, it would increase the initial pension.

This factor has been included in this model and its implementation is voluntary on the part of a beneficiary. That is, it is up to an individual and the economic need to cope with LTC costs. This proposed model has many practical implications as it can be implemented without much difficulty and even without any cost; coverage in private funded pension plans can be universalised. At present, these plans are designed with some hypotheses which assume a general mortality of the insured, but which do not particularise it in the case of being severely or highly dependent. For this reason, the inclusion of this factor, together with the mortality tables for dependent people, leads to the inclusion of the benefit without the need to increase costs or contributions.

It could be included automatically within the pension plan itself, but this would require:

- Accuracy and sufficient data on newly dependent people produced at each age, by degree of dependency, age and sex. Actuarial science requires a great deal of data from which to obtain significant probabilities. This would allow for the automatic incorporation of the transition probability of becoming a dependent. Thus, the application of the conversion factor would automatically adapt the expenditure to the life expectancy as a dependent resulting in a zero cost.
- Knowing the probability of a previous transition, a pension could also be designed that is totally independent and additional to the retirement pension; obviously, this would increase the final cost but would provide an opportunity to be financed progressively in the period of activity. Finding the optimum coverage would also come from determining and standardising the average cost at each age of the LTCs.

In the latter case, the automatic design with an independent benefit allows a distinction to be made, within the occupational pension scheme, between that part which may correspond to paying LTC expenses, as opposed to the pension itself as a substitute for or complement to income from work (deferred salary). 

## 6. Conclusions

In the course of this paper, a mechanism for the conversion of the old-age pension into a benefit for LTC support within a defined benefit pension scheme has been set out, specifically introducing an improvement to the benefits that a beneficiary currently receives. Unlike other approaches that finance LTC with contributions deducted from the retirement pension itself, this model proposes the adequacy of pension collection according to the specific mortality rates for a severely dependent person. In this way, once a person becomes dependent, they will receive their pension for less time, but it will be a higher amount. The aim is not to provide a solution for severely or highly dependent people, but rather to provide consistency in the pension that a retiree receives and to adapt it to their own life expectancy: the retiree receives a higher pension when they need it most.

## Figures and Tables

**Table 1:** Financial assets in employment pension funds (2019). Source: [27].

Country	Approximate Funds 2019 (USD Billion)	Structure
Australia	2077	Multi-employer funds, independent trustees and retail funds.
Canada	1924	Employer-sponsored, also industry and retail funds.
France	155	Unfunded industry-wide plans, employer-sponsored and insured retail funds.
Germany	502	Employer-sponsored also insured and unfunded.
Ireland	184	Employer-sponsored.
Italy	210	Employer-sponsored.
Japan	1400	(Excludes Japanese government pension fund.) Employer-sponsored, also retail funds.
Netherlands	1690	Employer- or multi-employer-sponsored.
Poland	48	Employer-sponsored.
South Africa	231	Employer- or multi-employer-sponsored.
Spain	43	Employer- or multi-employer-sponsored, also insured retail funds.
Switzerland	1047	Employer- or multi-employer-sponsored.
UK	3451	Employer-sponsored, multi-employer funds for minimum defined contribution benefits, also retail funds.
USA	29,196	Employer- or multi-employer-sponsored, also retail funds.
Total	42,158	

**Table 2:** Dependent excess mortality factors for the degree of severe and high dependency in Spain. Source: [84].

Factors	Men	Women
δ γ $x_{i}$	0.245	0.165
	1.135	1.09
	62.50	58.61
β	0.1142	0.0962

**Table 3:** Mortality rates and life expectancy of people born in 1970, values by sex and age. Source: Own work.

Age	$q_{x}^{m}\;$	$q_{y}^{m}$	$e_{x}^{m}$	$e_{y}^{m}$	q dxm	q dym	e dxm	e dym
20	0.009005	0.010674	85.4	92.5	0.009095	0.011724	54.3	48.6
25	0.000317	0.000285	81.4	88.7	0.000486	0.001895	49.9	44.5
30	0.000221	0.000162	76.5	83.8	0.000539	0.002627	45.0	39.9
35	0.000453	0.000202	71.6	78.9	0.001050	0.003963	40.2	35.5
40	0.001134	0.000280	66.8	74.0	0.002256	0.005997	35.4	31.3
45	0.001261	0.000275	62.2	69.1	0.003365	0.008909	30.9	27.3
50	0.001265	0.000325	57.6	64.2	0.005198	0.013246	26.4	23.5
55	0.001134	0.000340	52.9	59.3	0.008439	0.019416	22.2	20.2
60	0.001270	0.000395	48.2	54.4	0.014677	0.028025	18.2	17.2
65	0.001650	0.000518	43.5	49.5	0.025737	0.039513	14.6	14.6
70	0.002431	0.000740	38.9	44.6	0.054135	0.060454	11.6	12.5
75	0.003742	0.001048	34.4	39.8	0.085696	0.077951	9.1	10.8
80	0.005241	0.001447	30.1	35.0	0.124647	0.096128	7.3	9.4
85	0.007708	0.002037	25.9	30.2	0.165352	0.113471	6.1	8.4
90	0.010964	0.003079	21.9	25.6	0.201444	0.129209	5.3	7.6
95	0.017031	0.005315	18.2	21.0	0.231195	0.143675	4.7	7.0
100	0.025290	0.009638	14.7	16.6	0.255807	0.158651	4.4	6.3

**Table 4:** Main results for people born in 1970, values by sex and age. Source: Own work.

Age	$\lambda_{x}^{d}$	$\lambda_{y}^{d}$	q dxm−qx	q dym−qy	q dym−q dxm	exm−e dxm	eym−e dym
20	1.57	1.90	0.01%	0.10%	0.2891%	31.1	43.9
25	1.63	1.99	0.53%	5.64%	2.9023%	31.5	44.2
30	1.70	2.10	1.43%	15.17%	3.8768%	31.5	43.9
35	1.78	2.22	1.32%	18.58%	2.7743%	31.4	43.4
40	1.89	2.37	0.99%	20.39%	1.6587%	31.4	42.7
45	2.02	2.53	1.67%	31.43%	1.6474%	31.3	41.8
50	2.18	2.73	3.11%	39.76%	1.5481%	31.2	40.6
55	2.39	2.94	6.44%	56.13%	1.3008%	30.8	39.1
60	2.65	3.17	10.55%	70.01%	0.9094%	30.0	37.2
65	2.98	3.39	14.60%	75.35%	0.5352%	28.9	34.9
70	3.37	3.58	21.27%	80.70%	0.1167%	27.3	32.1
75	3.77	3.69	21.90%	73.42%	−0.0904%	25.3	29.0
80	4.10	3.71	22.78%	65.42%	−0.2288%	22.7	25.5
85	4.25	3.59	20.45%	54.70%	−0.3138%	19.8	21.8
90	4.15	3.35	17.37%	40.96%	−0.3586%	16.6	17.9
95	3.83	3.02	12.57%	26.03%	−0.3786%	13.4	14.0
100	3.37	2.63	9.12%	15.46%	−0.3798%	10.4	10.3

## Data Availability

The data presented in this study are available from the corresponding author on reasonable request.
